# Carnosinases, Their Substrates and Diseases

**DOI:** 10.3390/molecules19022299

**Published:** 2014-02-21

**Authors:** Francesco Bellia, Graziella Vecchio, Enrico Rizzarelli

**Affiliations:** 1 Institute of Biostructure and Bioimaging, CNR, viale A. Doria 6, 95125 Catania, Italy; E-Mail: francesco.bellia@cnr.it; 2 Department of Chemical Sciences, University of Catania, viale A. Doria 6, 95125 Catania, Italy; E-Mail: gr.vecchio@unict.it

**Keywords:** carnosine, carnosinase, dipeptide, metallopeptidase, biomarker

## Abstract

Carnosinases are Xaa-His dipeptidases that play diverse functions throughout all kingdoms of life. Human isoforms of carnosinase (CN1 and CN2) under appropriate conditions catalyze the hydrolysis of the dipeptides carnosine (β-alanyl-l-histidine) and homocarnosine (γ-aminobutyryl-l-histidine). Alterations of serum carnosinase (CN1) activity has been associated with several pathological conditions, such as neurological disorders, chronic diseases and cancer. For this reason the use of carnosinase levels as a biomarker in cerebrospinal fluid (CSF) has been questioned. The hydrolysis of imidazole-related dipeptides in prokaryotes and eukaryotes is also catalyzed by aminoacyl-histidine dipeptidases like PepD (EC 3.4.13.3), PepV (EC 3.4.13.19) and anserinase (EC 3.4.13.5). The review deals with the structure and function of this class of enzymes in physiological and pathological conditions. The main substrates of these enzymes, *i.e.*, carnosine, homocarnosine and anserine (β-alanyl-3-methyl-l-histidine) will also be described.

## 1. Introduction

Carnosinases are Xaa-His dipeptidases which belong to the metallopeptidase family M20 A of the metallopeptidase H clan, that play diverse functions throughout all kingdoms of life, ranging from a general role in the hydrolysis of late products of protein degradation to specific biochemical functions in protein maturation, tissue repair, and cell-cycle control [1]. The existence of an enzyme with the ability to hydrolyze the peptide bond in carnosine (β-alanyl-l-histidine, CAR) was first demonstrated by Hanson and Smith [2]. They named the enzyme carnosinase and classified it as a metal-ion-activated aminopeptidase, the activators being Mn^2+^ and Zn^2+^. More than 30 years later, Lenney *et al.* published the first molecular and functional characterization of human tissue [3] and serum carnosinase [4] Only in 2003 the gene sequences coding for the human serum (CNDP1 or CN1) and tissue (CNDP2 or CN2) carnosinase were identified [5]. This paper represents a milestone for the subsequent research about carnosinase. The same authors, a joint team of academic and industrial researchers, previously applied for two patents concerning the potential application of the human carnosinase isoforms, as indicative of the scientific and commercial interest that these enzyme might have.

The dyshomeostasis of carnosinase expression and activity cause several physiological dysfunctions and diseases, such as diabetes, ischemia, neurological diseases, wound healing, ocular diseases, *etc.* Most of them are due to the related dyshomeostasis of the main carnosinase substrate, carnosine.

Carnosine was the first peptide ever isolated from natural material by Gulewitsch and Amiradzibi [6]. The natural compound has been found to be widely distributed in several animal tissues. The wide range of protective properties (pH-buffering properties, antioxidant agent wound healing promoter, ion-chelating agent, especially for Cu^II^ and Zn^II^) makes carnosine of great interest for scientific and commercial uses, especially in the neuroprotection of oxidative stress-driven disorders [7]. 

Several reviews about the physiological role, the biological proprieties and the structural action of carnosine have been published [8], but none on the important proprieties of the carnosine-degrading enzymes, taking also into account that increased levels of carnosinase have been found in aging [9], whereas some neurological disorders, such as mild dementia, are characterized by reduced carnosinase levels [10].

This review deals with the structure and function of this class of enzymes in physiological and pathological conditions. Detailed features of carnosine and other histidine-containing dipeptides will be also described.

## 2. Carnosinase Substrates

**Figure 1:**
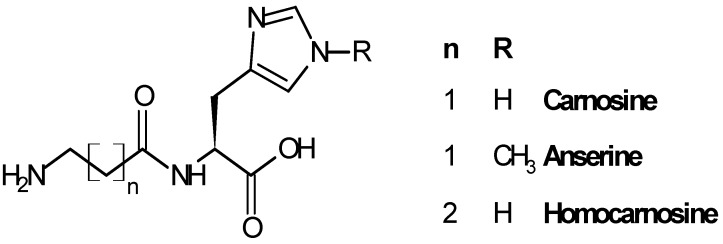
Structure of carnosine and related dipeptides.

Carnosine (β-alanyl-l-histidine, CAR), anserine (β-alanyl-3-methyl-histidine, ANS) and homocarnosine (γ-aminobutyryl-l-histidine, HCAR) are the three most representative compounds of the histidine dipeptides (Figure 1), widely distributed in mammals in different amounts, depending on the species and the tissue considered [11,12].

**Figure 2:**
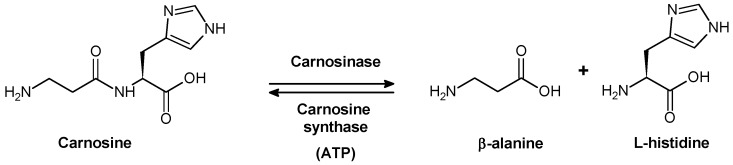
Synthesis and hydrolysis scheme of carnosine under physiological conditions.

CAR is synthesized from β-alanine and l-histidine by an ATP-dependent carnosine synthase enzyme (EC 6.3.2.11) (Figure 2), which has been detected in the extracts of chicken muscle and mouse brain [13,14]. Moreover, CAR synthase activity has been detected in glia cells – particularly in oligodendrocytes – derived from the rat brain [15] and shown to also catalyze the synthesis of homocarnosine. 

**Figure 3:**
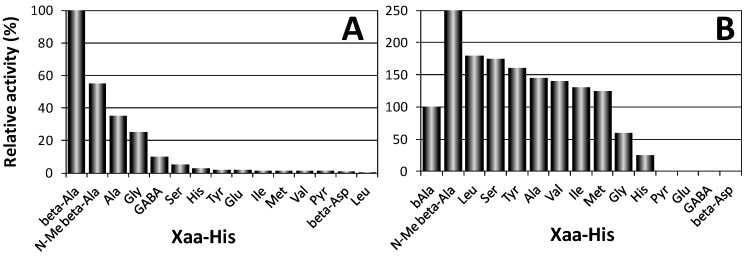
Hydrolysis activity of human CN1 (A) and CN2 (B) towards several Xaa-His dipeptides [5].

Two proteins encoded by different genes were shown to degrade carnosine in humans [5]. The first one (CN1, EC 3.4.13.20), also named “human serum carnosinase”, breaks down both carnosine and homocarnosine and is found in serum and brain tissue with high specificity (Figure 3A). The second one (CN2, formerly named “human tissue carnosinase”, EC 3.4.13.18) is a Mn^2+^ dependent cytosolic enzyme ubiquitously expressed in human tissues. This enzyme is now named “cytosol nonspecific dipeptidase,” because it does not degrade homocarnosine and exhibits a rather broad specificity toward various dipeptides (Figure 3B). Recently, it was reported that a mammalian gene with unknown function, ATPGD1, encodes an enzyme capable of synthesizing CAR and HCAR that has 15- to 25-fold higher catalytic efficiency with β-alanine than with γ-aminobutyrate [16].

ANS is synthesized from the enzymatic condensation of β-alanine with Nτ-methylhistidine through carnosine synthase in the brain and lens [17], and hydrolyzed to histidine by anserinase (Xaa-methyl-His dipeptidase, EC 3.4.13.5) in the brain and eye [18,19]. However, the main synthetic pathway of ANS occurs by N-methylation of carnosine, a reaction catalyzed by carnosine *N*-methyltransferase [20].

Carnosine is found in low concentrations (below 100 nM) in blood and the cerebrospinal fluid (CSF). HCAR concentration is also low in blood, while its concentration in the brain is 100-fold larger than that of carnosine [21]. Moreover, as for CAR, the cerebral content of HCAR is age-related, being three to six times larger in adults than in infants [22]. Thus, it has been suggested to be a precursor for the neurotransmitter GABA, hence serving as an important inhibitory neuromodulator in human neocortex [23].

The tissue content of histidine dipeptides are regulated by the activities and localization of carnosine synthetase and carnosinases. In humans, the diet has been proposed to represent the main source of histidine dipeptides [24]. The β-alanyl containing peptides (e.g., anserine and carnosine) are mainly found in mammalian skeletal muscle, whereas the γ-aminobutyryl-containing peptides (e.g., homocarnosine) are typical of the central nervous system. It remains unclear whether human muscle carnosine concentrations are stable [25]. Whereas it has been described that muscle carnosine concentrations are not affected by age, fitness or clinical status [26], a change in muscular carnosine concentrations probably caused by physical activity and reduced meat intake has been reported [27,28]. Also oral ingestion of β-alanine, the rate-limiting precursor in carnosine synthesis, increases carnosine synthesis in the muscle [29].

### 2.1. Biological Role of Carnosinase Substrates

The biological function of CAR is not fully understood, though it has been shown to possess a number of important properties including proton buffering capacity [30], antioxidant activity [31,32] and chelating ability [33,34,35]. Several studies have also demonstrated that CAR inhibits metastasis [36] and non-enzymatic glycosylation of protein [37], prevents the formation of protein-protein cross links by reacting with protein-carbonyl groups [38], delays diabetic deterioration [39], and influences glucose metabolism [40]. In the brain, CAR is primarily found in glial and ependymal cells [41,42], while peptide transporter 2 (PepT2) mediates the dipeptide transport in astrocytes [43]. In recent years, the occurrence of CAR and its analogues homocarnosine and anserine in CNS and their age-related alterations [44,45] suggested a therapeutic potential in neurodegenerative diseases [46,47]. Carnosine has been shown to be neuroprotective because of its capacity to counteract both oxidative [48] and nitrosative stress [49,50] related to several pathological conditions [51,52], including ischemia [53,54,55]. Recently, it has been demonstrated that CAR not only prevents the up-regulation of iNos and the induction of both HO-1 and Hsp-70 following strong nitrosative conditions [56], but also possesses NO free-radical scavenging ability and NO-trapping capacity in cell-free experiments [57,58]. Furthermore, CAR has been found to protect cells from amyloid-β induced toxicity [59] by its ability to inhibit protein aggregation by perturbing the H-bond network in and around the central hydrophobic cluster [60] and to avoid the formation of glycation end-products [61]. Interestingly, carnosine plasma levels have been found to be lower in patients with Alzheimer’s disease (AD) than in age-matched controls [62]. Exogenously administered CAR can cross the blood-brain barrier [63], but its efficacy as drug is a challenge due to the presence of carnosinase enzymes acting as endogenous dipeptidases [64].

Carnosine acts as a natural inhibitor of the angiotensin converting enzyme [65]. It restores erythrocyte deformability [66], and reduces the synthesis of matrix proteins such as fibronectin and collagen type VI of podocytes and mesangial cells [67]. Although the function of carnosine is better described, many studies showed that also anserine seems to have several protective functions. Similar to carnosine, anserine was showed to affect renal sympathetic nerve activity [68], reduces blood glucose [69], increases the contribution of the non-bicarbonate buffering action and decreases the bicarbonate buffering action in blood [70], acts as effective trans-glycating agents in decomposition of aldose-derived Schiff bases [71], protects neuronal cells against reactive oxygen species [72], shows dose-dependent angiotensin converting enzyme inhibitory activity [65], acts as peroxyl radical scavenger to protect the protein modification [73] and reacts as quencher of cytotoxic carbonyls [74]. Beside these similarities, there is some evidence that both dipeptides have also different functions. Whereas carnosine facilitates NO production in endothelial cells, anserine failed to increase NO production [68]. Treatment with carnosine, but not anserine, was able to significantly reduce infarct volume and improve neurological functions [75]. Further, an increase in the antitumor activity of doxorubicin [76] was only described for anserine. Beside these functions, the role of anserine in the kidney is not yet known and remains to be addressed.

Carnosine can reduce telomere shortening rate possibly by protecting telomeres from damage. Cells continuously grown in 20 mM carnosine exhibited a slower telomere shortening rate. The authors suggest that the reduction in telomere shortening rate and damages in telomeric DNA made an important contribution to the life-extension effect of carnosine [77].

Appreciable levels of l-carnosine have been found in transparent human lenses which are markedly depleted in mature cataracts [78]. The concentration of carnosine in transparent crystalline lenses detected was about 25 µM. At different stages of cataract development, the level of carnosine fell, reaching about 5 µM. Research with *N*-acetylcarnosine (NAC), an ophthalmic prodrug of l-carnosine, demonstrates that it is effective not only in preventing cataracts but also in treating them. NAC has been shown to improve vision by partially reversing the development of the cataract, thus increasing the transmissivity of the lens to light [79]. 

The clinical evidence has also been reported that carnosine, that finds its way into the aqueous humor and the crystalline lens through the topically applied NAC admixed with carboxymethylcellulose, is able to reduce telomeric attrition in the lens epithelial cells. This is due to a diminution in the oxidative stress, thus preventing the expression of the senescent phenotype of the lens epithelial cells [80]. 

Carnosine and its related-dipeptides are also efficient metal chelating agents (mainly for copper(II) and zinc(II)). Their copper complexes exhibit superoxide dismutase (SOD)-like activity [32], whereas Zn^II^-carnosine complex (called Polaprezinc) is effective for the repair of ulcers and other lesions in the alimentary tract [81,82]. Noteworthy, carnosine complexed to zinc(II) is able to induce the expression of important anti-oxidative stress proteins and enzymes, such as heme oxygenase 1 [83], Hsp72 [84], Hsp70 [85]. Although such studies have not been carried out in cerebral tissues, the involvement of these stress proteins in any type of both physiological and pathological oxidative stress-induced pathways [86], further emphasizes the crucial role of carnosine and its metal complexes in living systems.

Very recently, it has also been proven for the first time that carnosine is able to chelate the intracellular zinc(II) [87], whose mobilization was ROS-mediated. The scavenger activity of carnosine against ROS might represent a further protective effect on the reduction of ROS-mediated zinc(II) release.

For these reasons, it may act as neuroprotective agents in copper-dependent toxic conditions and as antioxidants in physiological and pathological conditions [7,88]. Indeed, the copper- and zinc- mediated neurotoxicity involved in several pathologies, such as amyotrophic lateral sclerosis, Alzheimer’s, Menkes’s, Parkinson’s, Pick’s and Wilson’s diseases [89,90], might be reduced or prevented by endogenous metal-chelating agents, such as CAR and its homologues [91,92]. Therefore, understanding the role of these endogenous compounds that are able to modulate copper availability and that have putative neuromodulatory and/or neuroprotective actions may help in the development of clinical approaches for the treatment of neuropathologies that involve metals and free radicals.

## 3. Human Carnosinases

The degradation of carnosine and related peptides has been firstly studied with extracts or partially purified enzyme preparations from porcine [2,93,94,95,96,97], murine [98,99], human [3] kidneys or rat brain [100]. A cytosolic form previously named tissue carnosinase (EC 3.4.13.18) was first isolated from porcine kidney by Hanson and Smith [2] in 1949. These authors and Rosenberg [95,96] observed that carnosine-degrading activity in hog kidney extracts was stabilized and activated by Mn^2+^ ions and strongly inhibited by metal-chelating agents. Later, Lenney *et al.* [93,94] described two metal-dependent carnosinases from the same material (porcine kidney) which exhibit distinct differences in their substrate specificity. These enzymes apparently do not contain essential sulfhydryl groups since they are not inhibited by *p*-chloromercuribenzoate, differently from the carnosine-degrading enzyme from brain, reported by Kunze *et al.* [82], and from human kidney, described by Lenney *et al.* [3].

The authors suggested, however, that “human tissue carnosinase” acts as a cytosolic nonspecific dipeptidase rather than a selective carnosinase based on its broad substrate specificity and the strong inhibition by bestatin [101]. A secreted form of human carnosinase was first described by Perry *et al.* [102] in patients with carnosinemia and was first purified from human placenta [103]. The enzyme was also isolated from human plasma and originally named human serum carnosinase (EC 3.4.13.20) [104]. It was distinguished from its cytosolic counterpart because of its particular distribution in human plasma and brain, its unique capability to degrade homocarnosine, and absence in non-primate mammals except for the Syrian golden hamster [4].

In 2003, Teufel *et al.* [5] revealed the nucleotide sequences of two novel genes CNDP1 and CNDP2, coding for human serum carnosinase and human tissue carnosinase (also called cytosolic nonspecific dipeptidase), respectively. The two types of CAR-degrading enzymes (CN1 and CN2) have been identified and characterized in humans and mice [5,105]. Sequence-based alignments of human CN1 and human CN2 with mouse CN2 show sequence identities of 53 and 91%, respectively.

Both purified recombinant enzymes have been characterized for their pH optimum of hydrolysis, substrate specificity, inhibitors, and the effects of metal ions on enzyme activity. The pH activity curve of CN1 protein showed a rather broad maximum between pH 7.5 and 8.5 essentially as previously described for human carnosinase [4]. A narrow pH optimum for carnosine degradation with a maximum around pH 9.5 has been reported for CN2. CN1 resulted insensitive to inhibition by bestatin [104], indicating that this enzyme represents the previously described serum carnosinase. In contrast, CN2 was sensitive to bestatin inhibition, required Mn^2+^ and dithiothreitol for enzymatic activity, and was inhibited by Zn^2+^ [5,105]. The substrate specificity of CN1 has been determined with Xaa-His dipeptides at pH 7.5 (Figure 3). Highest enzyme activity was found with carnosine, and only N-methylcarnosine, Ala-His, Gly-His, and homocarnosine served as substrate for this enzyme. Moreover, non Xaa-His dipeptides, as well as tripeptides containing histidine in central or C-terminal position, were not degraded by CN1 [5].

The substrate specificity of CN2 protein differed significantly. The enzyme did not hydrolyze carnosine but degraded dipeptides with good activity at pH 7.5 and in presence of 0.1 mM Mn^2+^. Only under non-physiological conditions (pH 9.5) carnosine served as substrate for CN2, but the relative dipeptidase activity with Xaa-His substrates like Leu-His, Ser-His, or Tyr-His was superior over carnosine degradation. Homocarnosine was not hydrolyzed at all. CN1 activity was completely abolished when three single point mutations (H133A, D166A, E201A), were introduced in CN1 with the aim of removing putative metal binding residues. 1,10-phenantroline also inhibited the enzyme in the low micromolar range [5]. 

Whereas at high concentrations (≥100 μM) cadmium activated CN1 protein, at low concentrations (0.1–3 μM) the metal ion strongly inhibited the enzyme. The saturation with Cd^2+^ ions had a strong effect on both the affinity and turnover of carnosine and homocarnosine degradation in the presence of 0.2 mM Cd^2+^, the kinetic parameters resulted 10-fold higher for both substrates as compared with conditions where cadmium was omitted. Other metal ions (Fe^2+^, Al^3+^, Co^2+^, Ni^2+^) with the exception of Cu^2+^, which strongly inhibited CN1 activity, had no effect on carnosine breakdown.

The selective activation of carnosinase by cadmium was described in the literature [5] as a criterion to distinguish between the different carnosine-splitting enzymes and can be used as evidence for a two-metal ion co-catalytic mechanism. Although the intrinsically bound metal ions in carnosinase are unknown, it has been suggested carnosinase is associated with Mn^2+^ and/or Zn^2+^ based on the homology to other enzymes of the M20 family of metalloproteases [5]. This hypothesis allows to explain why the activity of carnosinase varies in the presence of cadmium. At low cadmium concentrations Mn^2+^ or Zn^2+^ in a binding site (site 1) could be replaced with this ion, and catalysis of carnosine degradation is inhibited, whereas enzyme activity is reconstituted if both metal binding sites (1 and 2) are occupied by cadmium ions. Cytosolic non-specific dipeptidase can also be activated by Mn^2+^, Co^2+^, and Cd^2+^, which may explain why in previous studies [4,106] human carnosinase (secreted form) could not be distinguished from the cytosolic nonspecific dipeptidase. 

### 3.1. Biological Activities of Human Carnosinases

The biological function of both enzymes, although not yet well understood, may be quite different, as well. The expression pattern of CN1 differs in humans, mice, and rats: human CN1 is expressed mainly in the brain (particularly in pyramidal cells of the hypocampus) and liver, while CN1 is mainly expressed in the kidney and is not expressed in the brain of mice and rats [107].

**Figure 4:**
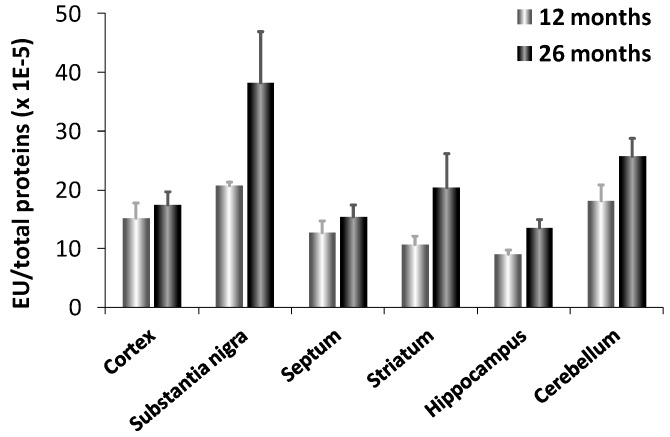
Enzyme activity of CN1 in different brain regions of adult (12 months) and senescent (24 months) rats [9].

Interestingly, fetal brain does not contain CN1 mRNA suggesting that CN1 gene expression is induced with age. This result explains why carnosinase activity was virtually undetectable in newborn humans and increased with age [108]. CN1 levels increase with age up to about 15 years in serum [104]. It has been also reported an increased expression and activity of carnosinase (CN1) in murine aging brain [9]. In aged animals, CN1 expression was 3-fold higher in substantia nigra compared to all other brain regions examined (cortex, septum, striatum, hippocampus and cerebellum), which showed comparable activities (Figure 4). Senescent rats, as compared to aged, exhibited a significant increase in CN1 expression in all brain regions, but the cortex. Notably, the maximum induction was observed in the substantia nigra and hippocampus, which are brain regions highly vulnerable to oxidant injury and aging effects. This may have important patho-physiological implications, in light of the possibility that an increased expression and activity of CN1, by decreasing carnosine levels can result in a significant decrease in antioxidative potential.

## 4. Other Aminoacyl-Histidine Dipeptidases

Anserinase was discovered by Jones [109], who found that anserine (β-alanyl-1-methylhistidine) was hydrolyzed by this enzyme in skeletal muscle of *G. callarias*. Anserinase is activated by bivalent metal ions and has broad specificity, with ability to hydrolyze many kinds of substrates such as *Na*-acetylhistidine, *N*-acetylmethionine, anserine, carnosine, homocarnosine, alanylhistidine, glycyl-leucine and leucylglycine [110]. Previously, it has been reported that this enzyme, purified from the brain of rainbow trout *Oncorhynchus mykiss* to apparent homogeneity, is a homodimeric protein with a subunit of 55 kDa [110]. It is commonly believed that anserinase is universally distributed in poikilothermic animals containing *Na*-acetylhistidine in their tissues [111]. Molecular identification of anserinase [112] revealed that it is a member of the M20 metallopeptidase subfamily, to which CN1 and CN2 belong.

Another two carnosine-cleaving enzymes, peptidase V and peptidase D, are known from bacterial species. Peptidase V belongs to the same peptidase subfamily (M20A) as those of the three above mentioned vertebrate enzymes, while peptidase D (EC. 3.4.13.3) belongs to a different subfamily M20C. Some crystal structure studies of these bacterial peptidases [113,114,115] as well as mouse CN2 [116] have shown that subunits of all the carnosine-cleaving M20 enzymes comprise a catalytic domain containing divalent metal ions including Zn^2+^ and Mn^2+^ at active site. Although enzyme activity of CN1 has never been detected in non-mammalian species as stated above, Partmann [117] reported a carnosine-cleaving enzyme activity in the skeletal muscle of European eel (*Anguilla anguilla*). No specific information describing the carnosine-cleaving enzyme(s) in eel muscle, however, is available. In fish, it has been known that skeletal muscles of two species of gadids, Atlantic cod (*Gadus morhua*) and haddock (*Melanogrammus aeglefinus*), are a rich source of Xaa-methyl-His dipeptidase, which can hydrolyze muscle anserine and is originally called ‘anserinase’ [118]. Thereafter, anserinase was demonstrated to have a broad specificity not only for many dipeptides including carnosine but also for several N-acetylated amino acids including *N*-acetyl-l-His, which is a physiological substrate for the enzyme in fish brain and eye [19,110]. A novel gene named ANSN, coding for anserinase, was identified from Nile tilapia (*Oreochromis niloticus*) [112].

**Figure 5:**
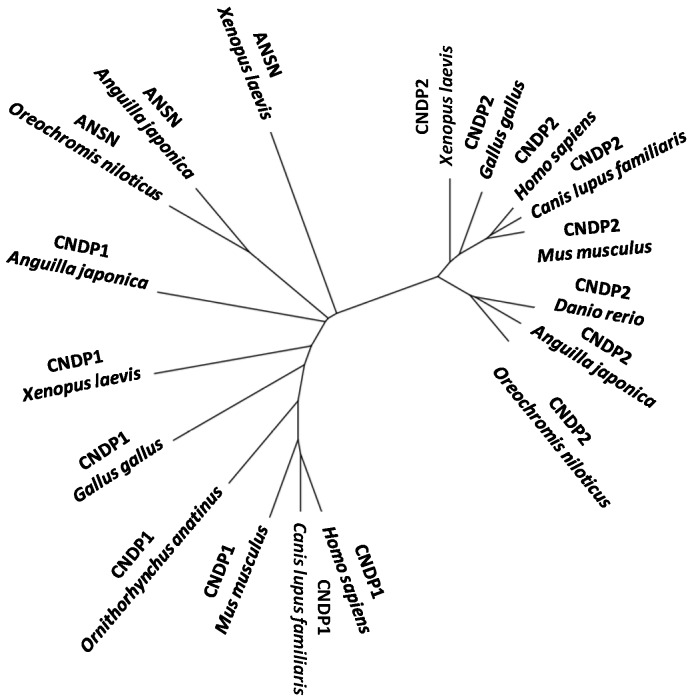
An unrooted phylogenetic tree of CNDP1, ANSN, and CNDP2 of M20A peptidase genes of vertebrates.

Recently [119], two different carnosine-cleaving enzymes were identified from the skeletal muscle of Japanese eel; one was carnosine dipeptidase I (encoded by CNDP1) having narrow specificity and another was Xaa-methyl-His dipeptidase (encoded by ANSN) having broad specificity. Both genes are strongly expressed in the liver, rather than in the skeletal muscle. This represents the first report on the identification of the dipeptidase encoded by CNDP1 from a non-mammal and may provide valuable insights into the phylogenetic history of the metallopeptidase subfamily M20A in vertebrate evolution (Figure 5).

## 5. Structural Features of Carnosinases

Based on the similarity in primary sequences, CN1 and CN2 have been classified as metallopeptidases belonging to the M20 family of clanMH. Of these, the crystal structures of PepV from Lactobacillus delbrueckii [120], CPG2 (carboxypeptidase G2) from *Pseudomonas spp*. [115] and PepD from *V. alginolyticus* [113,121] have been reported until now. The crystal structures of mouse CN2 complexed with bestatin together with Zn^2+^ and Mn^2+^ have also been obtained [116] (Table 1).

**Table 1:** Structural and functional properties of several carnosinases.

	Full name	Organism	Tissue	FW (KDa)	Activation	Optimal pH	Main substrate	Main Inhibitors
CN1	Serum carnosinase	*Homo sapiens*	Brain, serum, liver	56.8	Cd^2+^ citrate	7.5	Carnosine	Phenantroline
CN2	Cytosolic carnosinase	*Homo sapiens*	Ubiquitous	52.9	Mn^2+^	9.5	Xaa-His	Bestatin
ANSN	Anserinase	*Oncorhynchus mykiss*	Brain, eyes	55.0	Co^2+^, Zn^2+^		Anserine	Bestatin
PepV	Peptidase V	*Lactobacillus delbrueckii*	Cytoplasm	52.0			Carnosine	Phenantroline EDTA
PepD	Peptidase D	*E. Coli*	Cytoplasm	52.9	Co^2+^	9.0	Xaa-His	Metal chelators

PepV and CPG2 share 17% and 18% sequence identities with mouse CN2, respectively. Sequence-based alignment of PepD with proteins from the metallopeptidase H Clan shows low sequence identities and similarities in the range of 7%–20% and 13%–34%, respectively [114,115,122,123,124,125,126,127]. Nevertheless, sequence analysis revealed that putative active site residues for catalysis are conserved in PepD and related di-zinc enzymes in the M20 family [114,115].

**Figure 6:**
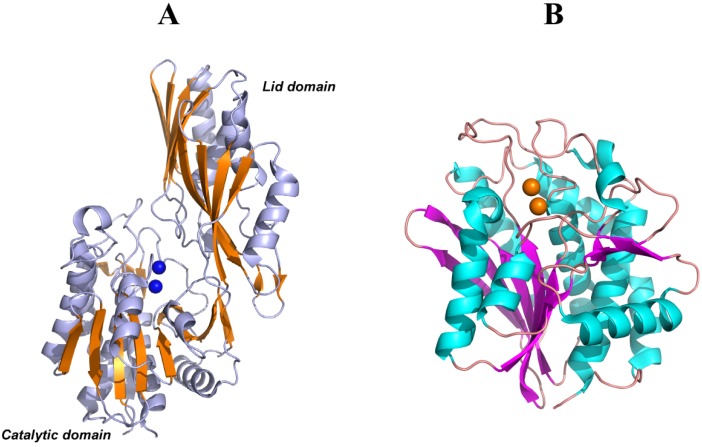
(**A**) Ribbon diagrams of PepV, in which β-sheets of the catalytic and lid domains are highlighted in yellow and the two catalytic zinc ions of the active center are represented as blue spheres. (**B**) Ribbon diagrams of AAP, in which β-sheets of the only catalytic domain are highlighted in magenta and the two catalytic zinc ions of the active center are represented as yellow spheres.

PepV, PepD and CPG2 are composed of two domains: one catalytic domain with two Zn^2+^ ions at the active center and one noncatalytic domain known as the lid domain or the dimerization domain. The dimerization domain of PepD and CPG2 provide the surface for the same interactions to form a homodimer structure, whereas PepV is present as a monomer due to the different structural features of the lid domain (Figure 6A). Furthermore, the crystal structure of a member of the M28 family of dinuclear zinc aminopeptidases from *Aeromonas proteolytica* (AAP) [122] is similar to the catalytic domain of PepV and CPG2. However, AAP does not have a non-catalytic domain and is present as a monomer in solution (Figure 6B).

Domain A of CN2 is structurally similar to the catalytic domains of PepV, PepD, CPG2, and AAP. This domain provides binding sites for two metal ions and a substrate to form the main part of the active site. Although the overall sequence homology was lower than 20%, metal ion ligands (His99, Asp132, Glu167, Asp195, and His445) and so-called “catalytic Glu” (Glu166) of CN2 were conserved in M20/M28 family proteins, including PepV, CPG2, and AAP, except that Asp195 is substituted with Glu in AAP.

**Figure 7:**
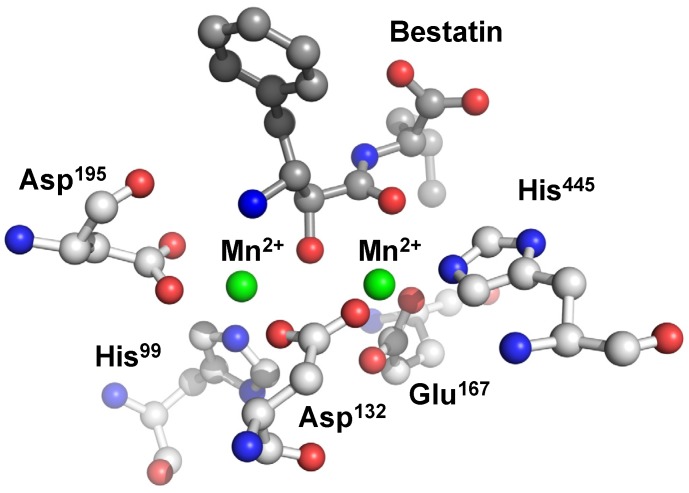
Active site of human CN2 crystallized with the inhibitor bestatin. The manganese(II) ions of the di-nuclear complex species are represented as green spheres.

The two metal ions are coordinated octahedrally by domain A residues and bestatin both in the Mn^2+^ and Zn^2+^ complexes. Metal 1 is coordinated by an imidazole nitrogen of His445, a carboxylate oxygen of Asp132, two carboxylate oxygens of Glu167, and two backbone carbonyl oxygens of bestatin. On the other hand, Metal 2 is coordinated by a carboxylate oxygen of Asp132, an imidazole nitrogen of His99, and two carboxylate oxygens of Asp195 together with a hydroxyl oxygen and the N-terminal nitrogen of bestatin. Each carboxylate oxygen of Asp132 coordinates to metals 1 and 2, respectively, to form a bridge-like structure between the two metals (Figure 7).

Similar to PepV, CPG2, and AAP, domain A of CN2 contains one *cis*-peptide bond between Asp132 and Asp133, this bond appears to be necessary to force the metal-bridging carboxylate such that it conforms to the correct geometry.

The metal ion selectivity of CN2 results completely different from those of M28 family metallopeptidases. CN2 requires Mn^2+^ selectively for its catalytic activity and is not activated by Zn^2+^, Cu^2+^, or Mg^2+^ [105], whereas all of the M28 family metallopeptidases are activated by Zn^2+^ [128]. Metal ion selectivity of AAP has been reported in detail; it is activated by Zn^2+^, Co^2+^, Ni^2+^, and Cu^2+^, whereas it is not activated by Mg^2+^ or Mn^2+^ [129]. A comparison of the active center of CN2 with those of M28 metallopeptidase family proteins reveals that the ligand residues and catalytic Glu are located in the same sites. The only remarkable difference between the active centers of CN2 and M28 peptidases is the orientation of the main chain oxygen atom of Asp195. This arrangement of Asp oxygen is unique to CN2 and PepV, whereas it does not exist in any structure of the M28 family reported. In the CN2-bestatin complex, it has a hydrogen bonding interaction with the N-terminal nitrogen atom of bestatin. This suggests that the N-terminal nitrogen of bound carnosine will probably also interact with the oxygen. Furthermore, the different orientation of the main chain oxygen atom of Asp195 could result in a differential selectivity of metal ions in addition to substrate specificity.

In the catalytic domain in PepD [113] His80, Asp119, Glu150, Asp173, and His461 are involved in metal binding, whereas Asp82 and Glu149 are necessary for catalysis. The N- and C-terminal ends are located on the top of the catalytic domain, opposite to the lid domain and the active site. Optimal activation of apo-PepD has been observed with various divalent metal ions, including Mn^2+^, Co^2+^, Ni^2+^, Cu^2+^, and Cd^2+^ [127]. Previous studies have shown that the addition of Co^2+^ ions to apo-PepD increases the enzymatic activity by a factor of ~1.4, compared with that of the wild-type PepD containing Zn^2+^. Moreover, Zn^2+^ did not inhibit Co^2+^-loaded PepD activity. Substitution of Zn^2+^ with Mg^2+^ resulted in an approximate 80% restoration of the optimal enzymatic activity. The di-zinc center is situated on the surface of the cleft between the catalytic and lid domains and, thus, it is solvent-accessible. The crystal structure of PepD also reveals that several functional residues interact and fix two zinc ions (Zn1 and Zn2). Zn1 is coordinated by one of the carboxylate oxygens of Asp119, Nε2 from His461, and a single putative water molecule bound by hydrogen bonding with the carboxylate group of Glu149. Zn2 is coordinated by Nε2 of His80, the other carboxylate oxygen of Asp119, and two carboxylate oxygens of Asp173. Asp119 is positioned as a bridging ligand between the two zinc ions. Notably, this residue is followed by an asparagine residue through a *cis* peptide bond as observed in many di-zinc-dependent enzymes of the M20/M28 family [115]. In PepV, the carboxylate oxygens of Glu154 point inward to Zn1, whereas in PepD the carboxylate oxygens of Glu150 point away from the Zn1. Thus, the role of metal ion binding for Glu150 in PepD remains ambiguous.

Currently, the crystal structures of the M20 family of proteins have been reported for two different (open and closed) conformations. When the protein is crystallized in a free form, the catalytic and lid domains are in an orientation that exposes the active site to bulky water; whereas when the protein is complexed with an inhibitor, a closed conformation has been observed. The structure of PepV complexed with an inhibitor showed that it has a closed conformation similar to that of CN2 complexed with bestatin [116].

In the PepV-inhibitor complex, a fixed “bridging” water molecule was found to be located between the two zinc ions and close to the carboxylate group of the catalytic Glu153, which corresponds to Glu149 of PepD and has been proposed as necessary for substrate hydrolysis. Upon binding of the substrate, the water molecule results placed between the zinc ions and the carbonyl residue of the scissile peptide bond. Then, an attacking hydroxyl ion nucleophile is able to be subsequently generated through activation of the water molecule by both the zinc ions and transfer of the resultant proton to the Glu153. Proximal to the Glu153 of PepV is the conserved metal-binding residue, Glu154, which utilizes its carboxylate oxygen to bind to the zinc ion. The carboxylate oxygen of Glu154 of PepV is directed toward the Zn1. Nevertheless, the structural analysis of PepD in an open conformation [113] revealed that the carboxylate oxygen of the corresponding Glu150 residue of PepD is directed away from the Zn1. It has been suggested that dipeptidases of M20 families can change their conformation from opened to closed during enzymatic catalysis [125]. The conformational change could be achieved by a movement of the catalytic and lid domains, as observed both for the PepD structure [113] and for the CN2 one [116]. It has been speculated that upon substrate binding, the PepD protein may change the metal ions’ coordination and/or its protein conformation [113,116]; the carboxylate oxygen of Glu150 would be subsequently swung toward Zn1 and would push the Glu149-bound water molecule toward Zn2, effectively bridging the water between the two zinc ions. However, the precise molecular interactions between the enzyme active site and the substrate or inhibitor still await final x-ray structure determination. The di-zinc binding amino acid residues, His80, Asp119, Glu150, and His461, are conserved among all of the proteins compared, but the Asp173 was found to be replaced by Glu in CPG2 and hACy1. This finding is consistent with the observation reported by Lindner *et al.* [125] that all homologs with proven aminopeptidase or dipeptidase specificity contain an aspartic acid, whereas a glutamic acid residue has been identified in the same position in Acyl1/M20 family members that exhibit either aminoacylase or carboxypeptidase specificity. 

Three residues, Asn217, His269, and Arg350 have been identified [114], within the lid domain of PepV, that are putatively involved in the substrate C-terminal and/or transition state binding through hydrogen bonding. Due to the different topology of the β-sheet order, a simple primary sequence alignment was not able to identify the corresponding residues in the lid domain of PepD, except for Arg369, which aligned with Arg350 of PepV. This residue also superimposed with Arg324, in the small domains of the dimeric CPG2. A structure-based sequence alignment has then been used to identify the other equivalent residues in PepD. A structure superimposition but inversed sequence order, in which the Asn217 and His269 residues of PepV superimposed with the Asn260 and His219 residues of PepD, has been found. Asn260 is conserved among PepV, CPG2, but is substituted by Thr in human CN1 and mouse CN2. Remarkably, the His219, Asn260, and Arg369 residues are located on the same side of the lid domain for both PepD dimers, whereas the corresponding residues are located on the opposite side of the lid domain of the same monomer for CPG2 and related dimeric proteins. Therefore, the Arg288 from the lid domain of one monomer interacts with the His229 and Asn275 from the lid domain of the other monomer in CPG2; in contrast, the Arg369 from the lid domain of the PepD monomer interacts with the Asn260 and His219 from the lid domain of the same monomer.

Site-directed mutagenesis experiments have been carried out to test the roles of these equivalent lid domain residues. Arg369 to Ala mutation resulted in complete loss of the enzymatic activity for hydrolyzing l-carnosine, whereas the Asn260 to Ala mutation decreased the catalytic activity to almost half. Interestingly, the His219 to Ala mutation did not affect the enzymatic activity significantly, yielding only a slight increase in activity of ~10% as compared with the wild-type PepD. In PepV, Arg350 results placed near the C terminus of the bound inhibitor (2.7 Å) but is too far away from the zinc ions, indicating a role in substrate binding but not in catalysis. The replacement of Arg with Ala might disrupt the hydrogen bond network between the Arg369 side chain and Asn260 Nδ with the carboxylate group of the substrate. In the case of PepV, Jozic *et al.* [114] have argued that domain flexibility is required to allow substrate access, in the case of PepV. A significant opening of the protein conformation would clearly benefit access of the peptides to the active site cavity. It is conceivable that even the whole lid domain might move away from its site to allow for easier substrate access and product egress. Therefore, although the Arg369 guanidinium side chain and the Asn260 Nδ within the active site of PepD are both away from the zinc ion, a conformational change between the open and closed states might contribute to the movement of both Arg369 and Asn260 upon substrate binding and subsequent transition state stabilization. Furthermore, binding of the His219 in PepD to the substrate likely persists during the conformational change between the open and closed states and contributes to transition-state stabilization through an electrostatic interaction between His219 and the free carboxyl group of the ligand, as shown in the PepV-inhibitor complex.

**Figure 8:**
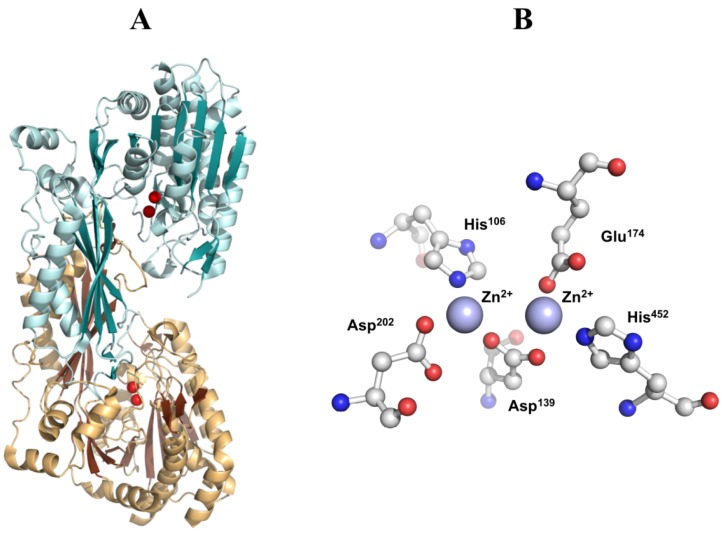
(**A**) Ribbon diagrams of dimer form of human CN1, in which the two catalytic zinc ions of the active center are represented as red spheres. (**B**) Active site of human CN1. The zinc(II) ions of the di-nuclear complex species are represented as violet spheres.

Recently, a crystal structure of CN1 complexed with zinc(II) was deposited in the Protein Data Bank. The protein has been crystallized in dimer form. The catalytic domain seems very similar to that reported for other carnosinases, although no paper has been published yet about these data. A Zn^2+^ ion is coordinated by the imidazole nitrogen of His106, the carboxylate oxygen of Asp202. The other zinc(II) ion is coordinated by a carboxylate oxygen of Glu174, an imidazole nitrogen of His452. Each carboxylate oxygen of Asp139 coordinates to both metal ions to form a bridge-like structure between the two metals (Figure 8).

**Figure 9:**
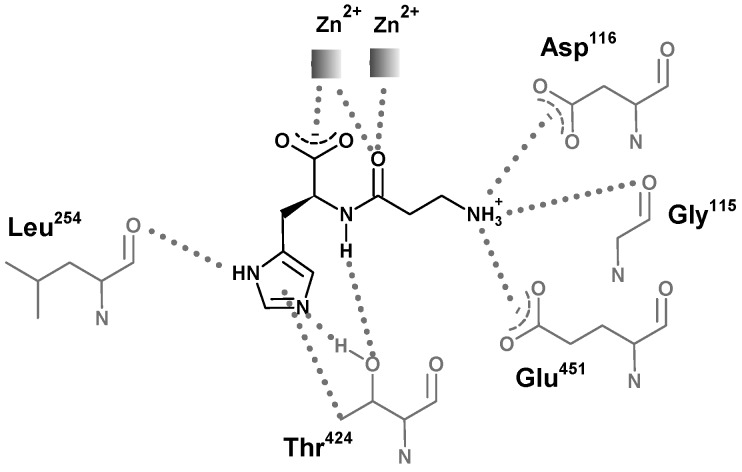
Structural representation of the simulated interaction pattern between human serum carnosinase CN1 and carnosine. The enzyme would bind the ammonium group, the carboxylate group, and the unsubstituted imidazole ring. The amido bond is simultaneously bound for recognition and polarized for catalysis [131].

The structures of carnosinases allow for a better knowledge of the molecular recognition of carnosine by carnosinase which might support the rational design of stable derivatives which conserve the peptide bond. Aldini *et al.* [130] suggested a putative complex between carnosine and human serum carnosinase and indicated the key interactions stabilized by the zinc ions with both the carboxylate and the carbonyl oxygen atom (Figure 9). This last contact should polarize the carbonyl group playing an essential catalytic role common of hydrolases. The protonated amino group of carnosine should interact with the carboxylates of Asp116 and Glu451, while the nitrogen atoms of the imidazole ring could elicit H-bonds with Leu254 and Thr424, thus providing a rationale for the specificity of serum carnosinase toward histidine-containing dipeptides and for the lower activity of serum carnosinase toward carnosine analogs with a substituted nitrogen in the imidazole ring, though anserine is, however, hydrolyzed by the enzyme.

## 6. Carnosinase-Related Diseases

Case studies of carnosinase deficiency have reported symptoms that include progressive mental deficiency (MD), non-progressive mental retardation, developmental delay, spastic paraplegia, seizures, neurosensory hearing loss, retinitis pigmentosa, and progressive dementia [132,133,134]. Reduced activity levels of CNDP1 have been found in other neurological disorders including Parkinson’s disease, multiple sclerosis, and following a cerebrovascular event [135,136]. On the contrary, CNDP2 is overexpressed in the substantia nigra in Parkinson’s disease [137]. Serum carnosinase levels have also been shown to decrease during cardiopulmonary bypass surgery [138] and it may be a neuroprotective mechanism, as carnosine may reduce neurotoxicity through its antioxidant capacity [92]. Several reasons have been suggested for altered carnosinase levels in healthy humans or with neurological disorders:

*i)*  disruption of the blood brain barrier (BBB) [136], probably due to cerebral ischemia [139], multiple sclerosis [140,141] and aggregation of Aβ in an AD mouse model [142];
*ii)*  damage to carnosinase-producing cells, though no relationship was found between size of the infarct and carnosinase activity [135];
*iii)*  genetic factors, as suggested by the fact that deletion distal to 18q21.3 was found in a child with serum carnosinase deficiency [143] (the gene for serum carnosinase is located on 18q22.3 [5]); moreover a locus on chromosome 18 was identified for familial AD in Caribbean Hispanics [144] and an association between the allelic variation of this gene and carnosinase activity has been shown [67]. In this study, patients with MD may have always had low carnosinase activity and then susceptible to dementia.
*iv)*  down-regulation of carnosinase mRNA, as shown for the AD marker neural thread protein (AD7CNTP). It is up regulated in the brains and CSF of patients with AD compared to controls [145].
*v)*  regular physical activity, associated with increased carnosinase activity and with a decreased risk of vascular dementia in women [146].

Many studies have focused on genetic association of CN1 with human diabetic nephropathy in the past several years after CNDP1 had been confirmed as a susceptibility locus [67,147,148,149,150,151,152,153,154,155,156,157,158]. The susceptibility to diabetic nephropathy is strongly associated with a polymorphism in the CNDP1 gene [67]. The CNDP1 polymorphism affects CN1 secretion. A low secretion is associated with smaller numbers (5-Leu) of a Leu-repeat in the signal peptide of human CNDP1. Diabetic patients homozygous for 5-Leu type of CNDP1 are protected against diabetic nephropathy, because carnosine acts as a protective factor against adverse effects of high glucose levels on renal cells. Recently, N-glycosylation was found essential for appropriate secretion and enzyme activity, in addition to the Leu-repeat polymorphism of CN1 [154].

The physiological mechanism for the protective effect of (CTG)5 homozygosity puts forward that (1) a CNDP1 genetic predisposition leads to low serum carnosinase activity [67]; (2) low carnosinase activity promotes higher concentrations of circulating carnosine; and (3) high circulating carnosine levels protect against hyperglycemia- induced cytotoxic metabolites resulting from oxidative stress and glycation. Although the association between the CNDP1 polymorphism and diabetic nephropathy have been confirmed in an independent study in European Americans [148], other studies did not show an association in type 1 diabetic patients [151] or showed that the association in type 2 diabetic patients is sex specific [153]. Inconsistent findings may be explained by differences in ethnicity [152] or, alternatively, by assuming that protection from diabetic nephropathy afforded by (CTG)5 homozygosity in CNDP1 may be masked by additional risk haplotypes [152,156].

Furthermore, while the expression of CN1 in transgenic db/db mice produced elevations in the blood glucose and hemoglobin A1c levels and a reduction in the blood insulin level, administration of CAR increased serum fasting insulin levels in db/db mice, and a significant correlation was observed between serum CAR levels and β-cell mass in the pancreas [40].

Marked alterations of renal carnosine metabolism in diabetic mice and their correlation with CN1 activity have recently been shown [159]. Renal CN1 activity is increased whereas tissue anserine concentrations are reduced ten-fold. Treatment with carnosine normalizes renal CN1 activity and renal anserine concentrations. Moreover, exogenous carnosine lowered blood glucose levels, proteinuria and renal vascular permeability. Increased CN1 activity in the diabetic mice might be the consequence of hyperglycemia due to the poor glucose control, in accordance with the finding that hyperglycemia enhances CN1 secretion and enzyme activity [154]. Carnosine treatment of db/db mice did not affect renal tissue carnosine concentrations, but normalized anserine levels. Because of anserine can be formed by methylation of carnosine via carnosine-*N*-methyl transferase [20,160], renal anserine increases in carnosine treated mice and accumulates, whereas exogenous carnosine is metabolized to anserine or hydrolyzed by CN1. In carnosine-treated diabetic mice, the decrease of CN1 activity may be due to lowered blood glucose levels that reduced *N*-glycosylation of CN1 [154] and/or the enzymatic inhibition of CN1 activity by anserine [158].

As a summary of all the carnosinase-related diseases, most of them are related to the effects that carnosinase overexpression has on the carnosine concentration. For this reason, diseases and disorders induced by carnosine dyshomeostasis (ischemia, neurological diseases, wound healing, ocular diseases, *etc.*), previously described in this review, may be related to variations of carnosinase levels.

### Carnosinase as a Biomarker

Among all the diseases, dementia has emerged as a major clinical, societal and economic problem, especially in industrialized societies. Dementia is also a common feature of many neurodegenerative diseases as Alzheimer’s, Parkinson’s and Huntington’s diseases [161]. Early and accurate diagnosis is desirable, as current therapies are most effective in the early stages and also because it could allow the cognitively aware patient to deal with future issues in medical care, safety, and legal matters.

Taking AD as an example, abundant evidence suggests the existence of a “preclinical” stage that can start 10–15 years before a subject can be diagnosed, where an individual is cognitively normal but is developing extensive pathological changes in the brain, particularly the build-up of amyloid plaques. It will therefore be important to have biomarkers that can identify individuals with preclinical AD or at the earliest clinical stages, in order to prevent or stem the synaptic and neuronal losses associated with cognitive impairment, seen the inadequacy of clinical examination to this purpose.

Numerous plasma and serum markers for dementia have been identified, but they lack the sensitivity and specificity needed for diagnosis and do not allow to monitor the progress of the disease, response to therapy or to predict outcome. Instead, cerebrospinal fluid (CSF) is a potentially rich source for AD biomarkers [162], as its composition is rapidly and directly influenced by the biochemical changes in the brain.

Indeed, a few CSF biomarkers have already been identified for clinical and even preclinical AD, the most studied proteins being Aβ42 (amyloid β), total tau, and phosphorylated tau species [163]. Among them, human serum carnosinase has also been proposed as a novel biomarker in CSF [163,164]. Although serum carnosinase activity has not shown significant differences between AD and controls, a difference between AD and mixed dementia (including vascular dementia) has been reported [134]. To our knowledge, an evaluation of plasma or CN1 concentrations in the context of AD has not yet been performed or reported. For this reason, further assessment of the potential of these and other proteins as candidate AD biomarkers in plasma or serum remains an important task for future studies.

Very recently, reduced levels of CNDP1 were observed in 8 out of 10 plasma specimens of patients with glioblastoma (GBM) [10]. Carnosine is also reported to have anti-growth property and has been discussed for its therapeutic potential against tumors including GMB [165]. Therefore, reduced levels of serum CNDP1 in the plasma of GMB patients may be important in the maintenance of carnosine levels and bioavailability of the dipeptide as a drug for GBM [138].

Human serum carnosinase has also been proposed as a novel specific biomarker for ischemic brain tissue damage during and after two different extracorporeal circulation systems [166]. Five minutes after sternum closure, the serum carnosinase activity in the minimized perfusion circuit group remained almost stable and was significantly higher as compared with the conventional circulatory perfusion bypass group, even after correction for hemodilution. This stability is due to a well-preserved oxygen capacity in the brain. 

## 7. Substrate Derivatives of Carnosine and Its Related Dipeptides

A large amount of scientific papers published of carnosine and carnosinase point out the paradox aspect of this biological system, *i.e.*, notwithstanding the beneficial role of endogenous and administrated carnosine, the enzymatic hydrolysis of the dipeptide is very rapid in the serum and in the intracellular environment of some tissues by CN1 and CN2, respectively.

Several attempts have been made to overcome this drawback, but almost all of them could be classified within two main strategies: the β-alanine implementation and the carnosine derivatization.

The first approach is being pursued in the field of the sport medicine with a number of results [167,168,169]. A large number of carnosine derivatives have been synthesized and structurally characterized [130,170].

The main goals reached by the carnosine functionalization have been: (a) avoiding or, at least, reducing the carnosinase hydrolysis; (b) conferring a lipophilic character with the aim to aid the BBB-crossing; (c) enhancing or, at least, maintaining the beneficial effects of the dipeptide; (d) counterbalancing the side effects of the grafted compound and (e) aiming at the targeted delivery.

Carnosine has been conjugated with several molecules, such as carbohydrates (cyclodextrins, trehalose, glucose, lactose, galactose) [34,171,172,173], antioxidants (Trolox, ascorbic acid) [174,175], drugs (l-Dopa) [176], physiological cofactors (lipoic acid, biotin) [177,178,179] and many aliphatic and aromatic moieties [180,181,182,183].

The carnosinase resistance has been reported for most of all the carnosine conjugates. The effect against oxidative stress, as well as the ability to bind several metal ions, mainly copper(II) and zinc(II), has largely been exploited [34,171,172,184].

One of the newest potential solutions consists in the use of the d-carnosine enantiomer (d-Car). d-Car is not hydrolyzed by the carnosinases and so its concentration could be maintained in the serum [183]. Moreover, it is able to cross the blood-brain barrier and to maintain the same quenching activity of L-carnosine (l-Car) *in vitro* [183]; hence, its use has been suggested for the treatment or prevention of oxidative stress-induced disorders [88,185]. Indeed, despite its behavior against oxidative- and nitrosative-induced damages, d-car is less bioavailable than l-Car because it is not recognized by PepT1 of the colon. Thus, as planned for l-Car, increasing attention has recently been paid to the functionalization of d-Car. Several hydrophilic [171,172] and lipophilic [185] molecules have been conjugated to d-Car with the aim of increasing the bioavailability of d-Car.

Chemical derivatization of homocarnosine and anserine has also been carried out not for enhancing or maintaining the beneficial effects of the dipeptide and aiming at the targeted delivery. HCAR and ANS were grafted on the upper and lower rims of β-cyclodextrin [186,187]. All these glycoside derivatives have higher antioxidant effects than those of the corresponding free histidine-containing dipeptides towards oxidation of human LDL and their copper(II) complexes catalyze the dismutation of superoxide anion (SOD-like activity) [188]. The HCAR-trehalose conjugate [184] maintains the copper(II) affinity of homocarnosine, shows SOD-like activity, inhibits the action of serum carnosinase on the hydrolysis of carnosine and, interestingly, it also delays the amyloid-β aggregation.

## 8. Concluding Remarks

Carnosinase and carnosine, as well as other aminoacyl-histidine dipeptidases and imidazole-containing dipeptides, are largely studied in terms of their structure, function and alteration in pathological conditions. Their localization mainly in cerebral tissues has made these molecular systems very attractive for the scientific community because understanding their physiological functions might reasonably be crucial for the development of new strategies to cure or prevent widespread brain-related diseases. The proposal of serum carnosinase as a promising biomarker of AD in CSF [164] represent an important starting point in this direction.

Diabetes is another important disease in which the carnosine-carnosinase system may play a crucial role. Therefore, CN1 is a potential target for pharmacological interventions aimed at manipulating glucose metabolism. Since only approximately one-third of the entire human population is homozygous for the “low-risk” CNDP1 allele, this approach has the potential for a broad applicability to diabetic patients [40].The paradox aspect of the carnosine-carnosinase system, *i.e.*, the rapid hydrolysis of the dipeptide in human serum despite the beneficial effects of administrated carnosine well ascertained in physiological and pathological conditions, needs to be overcome and the chemical derivatization has been proposed as a promising strategy to increase the bioavailability of this dipeptide.
